# Toll-Like Receptor 9 Mediated Responses in Cardiac Fibroblasts

**DOI:** 10.1371/journal.pone.0104398

**Published:** 2014-08-15

**Authors:** Ingrid Kristine Ohm, Katrine Alfsnes, Maria Belland Olsen, Trine Ranheim, Øystein Sandanger, Tuva Børresdatter Dahl, Pål Aukrust, Alexandra Vanessa Finsen, Arne Yndestad, Leif Erik Vinge

**Affiliations:** 1 Research Institute of Internal Medicine, Oslo University Hospital Rikshospitalet, Oslo, Norway; 2 Faculty of Medicine, University of Oslo, Oslo, Norway; 3 Center for Heart Failure Research, University of Oslo, Oslo, Norway; 4 Section of Clinical Immunology and Infectious Diseases, Oslo University Hospital Rikshospitalet, Oslo, Norway; 5 K.G. Jebsen Inflammatory Research Center, University of Oslo, Oslo, Norway; 6 Department of Cardiology, Oslo University Hospital Rikshospitalet, Oslo, Norway; 7 K.G. Jebsen Cardiac Research Center, University of Oslo, Oslo, Norway; University Hospital Medical Centre, Germany

## Abstract

Altered cardiac Toll-like receptor 9 (TLR9) signaling is important in several experimental cardiovascular disorders. These studies have predominantly focused on cardiac myocytes or the heart as a whole. Cardiac fibroblasts have recently been attributed increasing significance in mediating inflammatory signaling. However, putative TLR9-signaling through cardiac fibroblasts remains non-investigated. Thus, our aim was to explore TLR9-signaling in cardiac fibroblasts and investigate the consequence of such receptor activity on classical cardiac fibroblast cellular functions. Cultivated murine cardiac fibroblasts were stimulated with different TLR9 agonists (CpG A, B and C) and assayed for the secretion of inflammatory cytokines (tumor necrosis factor α [TNFα], CXCL2 and interferon α/β). Expression of functional cardiac fibroblast TLR9 was proven as stimulation with CpG B and –C caused significant CXCL2 and TNFα-release. These responses were TLR9-specific as complete inhibition of receptor-stimulated responses was achieved by co-treatment with a TLR9-antagonist (ODN 2088) or chloroquine diphosphate. TLR9-stimulated responses were also found more potent in cardiac fibroblasts when compared with classical innate immune cells. Stimulation of cardiac fibroblasts TLR9 was also found to attenuate migration and proliferation, but did not influence myofibroblast differentiation *in vitro*. Finally, results from *in vivo* TLR9-stimulation with subsequent fractionation of specific cardiac cell-types (cardiac myocytes, CD45+ cells, CD31+ cells and cardiac fibroblast-enriched cell-fractions) corroborated our *in vitro* data and provided evidence of differentiated cell-specific cardiac responses. Thus, we conclude that cardiac fibroblast may constitute a significant TLR9 responder cell within the myocardium and, further, that such receptor activity may impact important cardiac fibroblast cellular functions.

## Introduction

Toll-like receptors (TLRs) belong to a subfamily of germline-encoded pattern recognition receptors that recognize conserved pathogen associated molecular patterns (PAMPs), as well as danger associated molecular pattern (DAMPs), eliciting innate immune responses during non-sterile and sterile inflammation, respectively [1]. TLR9 is unique in its ability to respond to microbial DNA, more specifically through recognition of cytosine-phosphate-guanine (CpG) motifs which are abundant in bacterial DNA [2]. This, in addition to its localization in endosomes, allows TLR9 to discriminate between foreign pathogen derived DNA and endogenous DNA [2], [3]. However, recent studies have demonstrated sterile activation of TLR9 through the release and subsequent activation by CpG-containing mitochondrial DNA [4], [5].

All TLRs are expressed in classical innate immune cells such as monocytes, macrophages and dendritic cells (DC) [6], but expression of TLRs has also been found in non-immune cells in several organs, including the myocardium [7], [8]. Although activation of the innate immune system is an important feature in heart disease [9] little is known about the significance of cardiac TLR9 in this setting. Stimulation with bacterial DNA has been shown to activate TLR9 in cardiac myocytes (CM) and cause cardiac dysfunction [10]. Also, TLR9-signaling seems to be nodal in experimental viral myocarditis [11], and activation of cardiac TLR9 prior to cardiac ischemia and reperfusion has been shown to limit subsequent myocardial damage and improve cardiac function [12]. However, results from studies investigating the role of TLR9 in pressure-overload induced heart failure are ambiguous, as both beneficial and harmful effects of TLR9-activation have been shown [5], [13].

Most studies on cardiac TLR9 have focused on the heart as a whole or solely on CM. However, although CM makes up for the bulk volume of the heart, they are outnumbered by cardiac fibroblasts (CF) [14]. Furthermore, CFs are proposed to function as sentinel cells in response to chemical and mechanical signals of the myocardium [15], and has recently been shown to convey potent immunomodulatory and inflammatory properties [16]. Despite this, the role of CFs in cardiac TLR9-signaling is in large non-investigated. Thus, the aim of this study was to investigate TLR9-signaling in CFs, *in vitro* and *in vivo*, and furthermore characterize functional cellular implications of said TLR9-effect.

## Methods

### Ethics statement

All animal experiments were approved by the Norwegian Animal Research Committee and were in accordance with the “Principle of laboratory animal care” (NIH publication No. 86-23, revised 1985).

### Isolation and maintenance of murine primary cells

Adult C57BL/6 mice (both male and female) were anaesthetized with >5% isoflurane gas and <95% O_2_ in a gas chamber before hearts were explanted. Cardiac cells were isolated from the hearts by retrograde perfusion and enzymatic digestion using collagenase type 2 (Worthington Biochemical Corporation, Lakewood, NJ, USA), as previously described [17]. CMs were collected by centrifugation at 20× *g* for two minutes. The subsequent pellet was resuspended once and centrifuged a second time to ensure purity. The supernatant was cleared for CMs by an additional centrifugation at 20× *g* for two minutes before collecting cells by centrifugation at 300× *g* for 5 minutes. For subsequent cell-culture experiments non-CM pellets were resuspended in 12 ml DMEM (Gibco, Carlsbad, CA, USA) containing 10% fetal calf serum (FCS) (PAA Laboratories, Pasching, Austria) and 5 U penicillin/ml and 50 µg/ml streptomycin (Sigma-Aldrich, St.Louis, MO, USA), seeded onto 75 cm^2^ culture bottles and incubated with 5% CO_2_ at 37°C. The cells were propagated through one passage before experiments were performed.

The isolation, propagation and differentiation of bone marrow derived macrophages and DC were performed as previously described [18], [19]. In brief, cells were collected by flushing out bone-marrow contents of tibias and femurs (from the same mice as used for isolating CFs) using RPMI 1640 (PAA Laboratories) supplemented with 10% FCS and 50 U penicillin/ml and 50 µg/ml streptomycin (Sigma-Aldrich). Cells were centrifuged (800× *g*/5 minutes/4°C) and pellets were resuspended in 20 ml RPMI medium containing 10 ng/ml macrophage-colony stimulating factor (M-CSF) or 20 ng/ml granulocyte macrophage-colony stimulating factor (GM-CSF) (R&D Systems, Minneapolis, MN, USA) for differentiation into macrophages or DC, respectively. The cells were incubated with 5% CO_2_ at 37°C with replenishment of medium containing differentiation factors every second day for one week to ensure optimal differentiation.

### RNA extraction and quantitative PCR

Total RNA from tissue and cells were isolated using RNeasy Mini Columns (QIAGEN, Hilden, Germany). cDNA was synthesized using High Capacity cDNA Reverse Transcription Kit (Applied Biosystems, Carlsbad, CA, USA). Quantification of gene expression was performed by quantitative real-time PCR, using Power Sybr Greene Master Mix (Applied Biosystems) and sequence specific PCR primers (Table 1). Gene expression of the housekeeping gene glyceraldehyde 3-phosphate dehydrogenase (GAPDH) confirmed equal loading.

**Table 1:** Primer sequences used in qPCR assays.

Target	Species	Sequence (5′→3′)	Acc.nr
**18S**	Mouse/Human	(+)-CGGCTACCACATCCAAGGAA	NR_003286
		(−)- GCTGGAATTACCGCGGCT	
**β-actin**	Mouse/Human	(+)- AGGCACCAGGGCGTGAT	NM_001101
		(−)- TCGTCCCAGTTGGTGACGAT	
**CD3γ**	Mouse	(+)-CGAGGCACGTATCAGTGTCAA	NM_009850
		(−)-TCAATGCAGTTTTCACACATTCTG	
**CD45**	Mouse	(+)-GGTGCCAGCCTCACAACTCT	NM_011210
		(−)-CCAAACATGGCAGCACATGT	
**Collagen IaI**	Mouse	(+)-CCTGAGTCAGCAGATTGAGAACA	NM_007742
		(−)-TCGATCCAGTACTCTCCGCTCT	
**CXCL2**	Mouse	(+)-CACCCAAACCGAAGTCATAGC	NM_008176
			
		(−)-AATTTTCTGAACCAAGGGAGCTT	
**GAPDH**	Rat/Mouse/Human	(+)-CCAAGGTCATCCATGACAACTT	NM_008084
		(−)-AGGGGCCATCCACAGTCTT	
**TLR9**	Mouse	(+)-TCCATCTCCCAACATGGTTCTC	NM_031178
		(−)-GCCAGCACTGCAGCCTGTA	
**TNFα**	Mouse	(+)-AGACCCTCACACTCAGATCATCTTC	NM_013693
		(−)-CCACTTGGTGGTTTGCTACGA	
**Troponin T2**	Mouse	(+)-TCGACCTGCAGGAAAAGTTCA	NM_011619
		(−)-TGGAGACTTTCTGGTTGTCATTGA	

GAPDH was chosen as housekeeping gene.

### ELISA analysis of TLR9-stimulated release of cytokines and chemokines

Analysis of CXCL2, Tumor Necrosis Factor α (TNFα), CXCL10 and Interferon (IFN) α/β levels in conditioned culture medium were performed using commercially available ELISA kits (DuoSet Mouse CXCL2/MIP-2 (IL-8), Mouse TNFα, Mouse CXCL10/IP-10/CRG-2, R&D Systems, and VeriKine Mouse Interferon alpha/beta ELISA kit, PBL Biomedical Laboratories, Piscataway, NJ, USA) according to the specifications of the manufacturer.

### *In vitro* assessments of pharmacological characteristics of TLR9-stimulated responses

Cells were transferred to 12-well cell culture plates (Macrophages and DC: Nunc, Thermo Fisher Scientific Inc, Watham, MA, USA, and CF: Corning Incorporated, Corning, NY, USA) and used at a density of 80%. At experimental start, cells were washed with PBS and incubated for 3 hours with 500 µl of medium (DMEM or RPMI) containing 1% FCS. Temporal profiles of TLR9-stimulated responses in macrophages, DC and CFs were obtained by incubating cells with various murine TLR9 agonists (oligodeoxynucleotide [ODN] 1585 class A [CpG A], ODN 1668 class B [CpG B]; and ODN 2395 class C [CpG C], Invivogen, San Diego, CA, USA) for different time points (10 minutes through 24 hours) before analysis of CXCL2, TNFα, IFNα/β and CXCL10 release. Dose-response relationships were obtained by stimulating cells with CpG A, B or C (10 ng/ml through 10 µg/ml) for 18 hours and subsequent analysis of CXCL2 and TNFα. To investigate TLR9 specificity, cells were added single doses of CpG B or the TLR4 agonist LPS (Invivogen), together with the murine TLR9-antagonist ODN 2088 (Invivogen) or chloroquine diphosphate (Sigma-Aldrich) at final concentrations of 100 ng/ml, 10 ng/ml, 10 µg/ml and 5 µg/ml respectively, for 18 hours before analysis of CXCL2 and TNFα.

Cell counts of CFs, bone marrow derived macrophages and DC, from single wells of 12-well cell culture plates, were obtained by using Countess Automated Cell Counter (Life Technologies Corporation, Carlsbad, CA, USA) according to the specification of the manufacturer, and further used to normalize experimental data in regard to number of viable cells. No significant differences in cell viability between CFs, DC or macrophages could be detected.

### Assays of CF proliferative/migratory responses and myofibroblast differentiation upon TLR9 activation

Scrape-wound assay: CFs were grown as monolayer until 100% confluency in 6-well plates (Corning Inc), washed with PBS before incubation in serum-free medium (DMEM) for 5 hours before applying a standardized distortion of the monolayer using a p-10 pipette tip. Cells were subsequently washed with PBS and added culture medium (DMEM with 0.1% FCS) with CpG B (0.1 µg/ml) and/or ODN 2088 (1 µg/ml). The re-colonization of the distorted surfaces were documented by photography at different time points (1 and 18 hours after cell distortion) using a Motic AE2000 inverted microscope (Motic, Xiamen, China). Subsequent analysis of re-colonization rate was performed using ImageJ processing and analysis software.

Proliferation assay: Assessments of CF-proliferation upon TLR9-activation was performed using the Cell Proliferation ELISA, BrdU (chemiluminescent) assay (Roche Applied Science, Penzberg, Germany), according to instructions supplied by the vendor. In brief, CFs were seeded onto 96-well cell culture plates with a density of 50 cells/mm^2^. At experimental start, cells were washed with PBS before subsequent incubation in serum-free DMEM for 5 hours. After five hours cells were added CpG B (0.1 µg/ml) and/or ODN 2088 (1 µg/ml) in DMEM with FCS (0.1%) and incubated over night. The following day BrdU labeling agent was added, and cells were incubated for additional 5 hours before medium was removed and cells were fixed. An anti-BrdU-POD antibody was subsequently added to the fixed cells for one hour, before washing and developing by adding substrate solution. Degree of chemiluminescence was quantified using a microplate luminometer (Victor^3^ 1420-012 Multilabel Counter, Perkin Elmer, Waltham, MA).

Differentiation assay: CFs were grown as monolayer until 80% confluency in 6-well plates (Corning Inc). The cells were washed with PBS before incubation with serum-free medium (DMEM) overnight. The following day cells were washed with PBS and supplied with serum-free culture medium, before adding CpG B (100 ng/ml) and/or ODN 2088 (1 µg/ml). Separate wells were added TGFβ (2.5 ng/ml) (R&D Systems) as positive control. After 24 hours of stimulation, cells were harvested and RNA was isolated. mRNA levels of αSMA were measured as a surrogate marker for CF-differentiation.

### Cell separation

Male C57BL/6 mice were given i.p. injections of 100 µl/50 µg CpG B or 100 µl vehicle 24 hours prior to euthanization by extraction of hearts, followed by enzymatic release of cardiac cells (as described above) and subsequent cell-separation. Cell-separation was performed using MACS Technology (Miltenyi Biotec GmbH, Bergisch Gladbach, Germany) in accordance to specifications supplied by the manufacturer. Briefly, pellets of the non-CM fraction were re-suspended in buffer containing phosphate-buffered saline (PBS; Sigma-Aldrich), 0.2 mg/ml DNAse I (Roche Applied Science, Penzberg, Germany), 0.5% low-endotoxin BSA (Sigma-Aldrich) and 1 mM EDTA (Sigma-Aldrich), filtered through pre-separation filters (30 µm, MACS, Miltenyi), before incubation with CD45 MicroBeads (MACS, Miltenyi) at 4°C for 15 minutes. After incubation, labeled cells were isolated by magnetic retraction through an MS column (MACS, Miltenyi), washed and subsequently eluted. Similar procedure was applied on the resultant cell-fraction (non-CM/non-CD45^+^) using CD31 MicroBeads (MACS, Miltenyi). Final fractions (CD45^+^, CD31^+^ and CF-enriched fraction [non-CM/non-CD45^+^/non-CD31^+^]) were collected by centrifugation, and all cell-pellets were snap-frozen in liquid nitrogen and stored at −80°C before further analysis.

### Statistical analyses

Data were analyzed using GraphPad Prism 5 (GraphPad, San Diego, CA, USA). All values are presented as mean ± SEM. For non-parametric testing the Mann-Whitney U test was used for comparison of two groups, while Kruskal-Wallis test was used *a priori* when more than two groups were compared. Probability values of *p*<0.05 (2-sided) were considered statistically significant.

## Results

### TLR9 signaling properties in CFs

**Figure 1:**
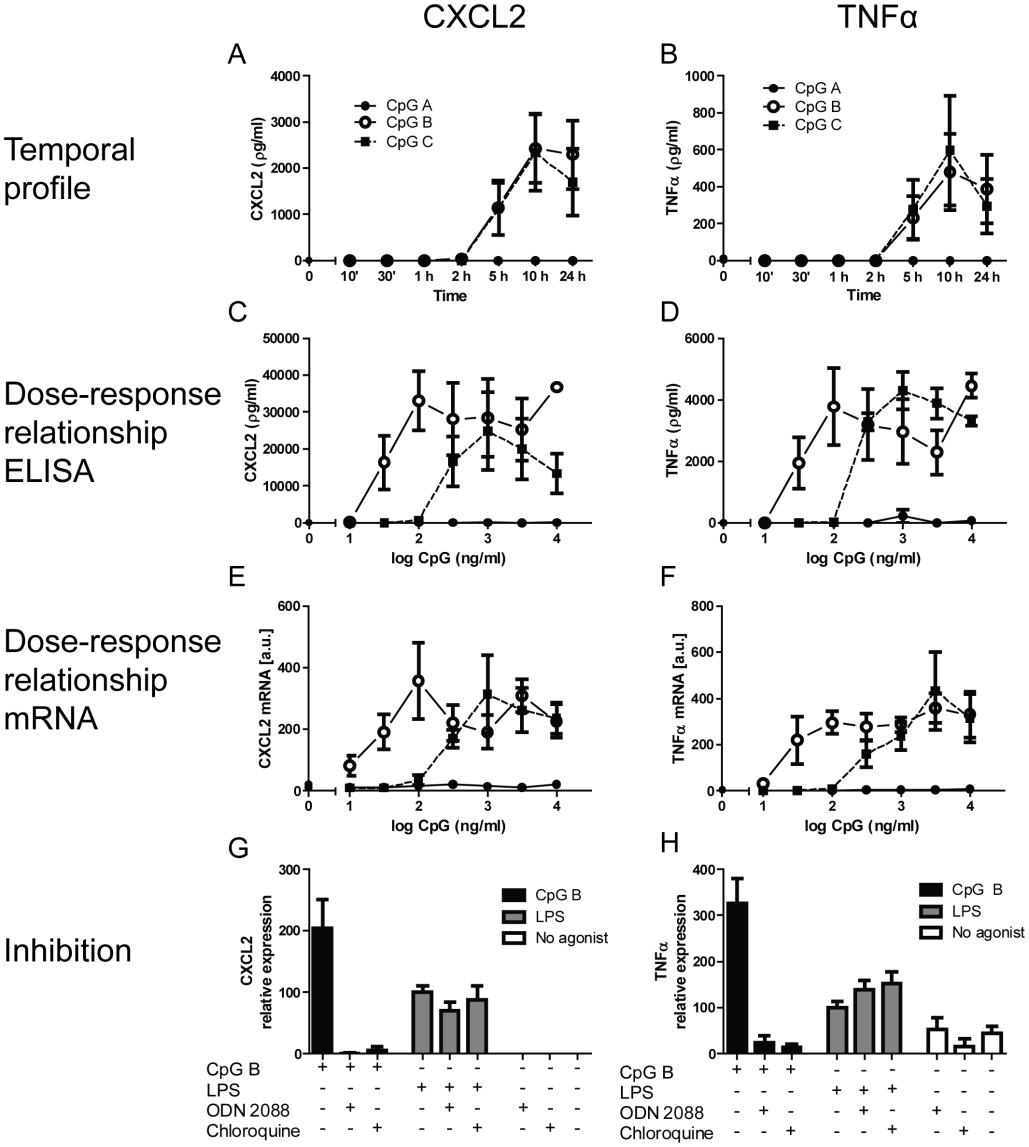
TLR9-stimulated responses in cardiac fibroblasts. **Temporal profiles** (10 min, 30 min, 1 h, 2 h, 5 h, 10 h and 24 h) of (all at 100 ng/ml) CpG A (dots), CpG B (open circles) or CpG C (squares)-stimulated release of CXCL2 (panel A) and TNFα (panel B) in murine cardiac fibroblasts (CF). Each data point represents the mean ± SEM of 3 separate experiments. **Dose-response relationships** of CpG A (dots), CpG B (open circles) and CpG C (squares) –stimulated release of CXCL2 (panel C) and TNFα (panel D) at 18 hours. Each data point represents the mean ± SEM of 6 experiments. Expression levels of CXCL2 (panel E) and TNFα (panel F) were analyzed by qPCR. Each data point represents the mean ± SEM of 4 experiments. **TLR9 specificity studies** were performed on murine CFs exposed to CpG B (100 ng/ml) or LPS (10 ng/ml) with or without the presence of ODN 2088 (10 µg/ml) or chloroquine diphosphate (5 µg/ml). Levels of CXCL2 (panel G) and TNFα (panel H) were analyzed after 18 hours. Each data point represents the mean ± SEM of 3 experiments.

TLR9-signaling pathways are divided into those culminating in activation of nuclear factor κB (NFκB), a type 1 IFN-response, and/or mitogen-activated protein kinase (MAPK) pathways [20]. Which of the specific signaling pathways are activated is determined by the nucleotide composition of the stimulating DNA, as well as the responder cell itself [21], [22]. As for the former, three major classes of TLR9-activating CpG ODNs exist (CpG A, B and C). In order to evaluate responses upon TLR9 activation in CFs we did a series of experiments stimulating cells with CpG A, B and C and subsequent measuring parameters reflecting NFκB activation (CXCL2), activation of MAPK/NFκB (TNFα) and type I IFN (INFα, IFNβ and CXCL10). As shown in Fig. 1A–B, a robust release of both CXCL2 and TNFα could be demonstrated after stimulation of CF TLR9 for 10 minutes through 24 hours. However, only CpG B and C but not CpG A, led to detectable TLR9 mediated responses. In addition, dose-response relationships clearly outlined CpG B as the most potent as compared to CpG C (Fig. 1C–D). These data were substantiated in separate experiments measuring TLR9 stimulated expression levels of mRNAs encoding CXCL2 and TNFα (Fig. 1E–F). Stimulating CFs with CpG ODNs may in theory activate DNA-sensing receptors other than TLR9. Data in Fig. 1G–H demonstrate that the effects seen in our experiments are likely due to activation of endogenous TLR9 as both a specific TLR9-antagonist (ODN 2088) as well as an inhibitor of endosomal acidification (a requirement for TLR9 activation); chloroquine diphosphate, completely abolished CpG-mediated responses (Fig. 1G–H). This notion was further substantiated as neither chloroquine diphosphate, nor ODN 2088, affected TLR4-stimulated production of CXCL2 and TNFα (Fig. 1G–H). In contrast to the observed TLR9 stimulated NFκB and MAPK/NFκB responses, we were not able to detect type I IFN responses with either of the CpGs (data not shown). This observation was substantiated by the findings of barely detectable IFNβ transcript levels under the same experimental conditions.

### CpG-stimulated responses in CFs in comparison to responses in dendritic cells and macrophages

**Figure 2:**
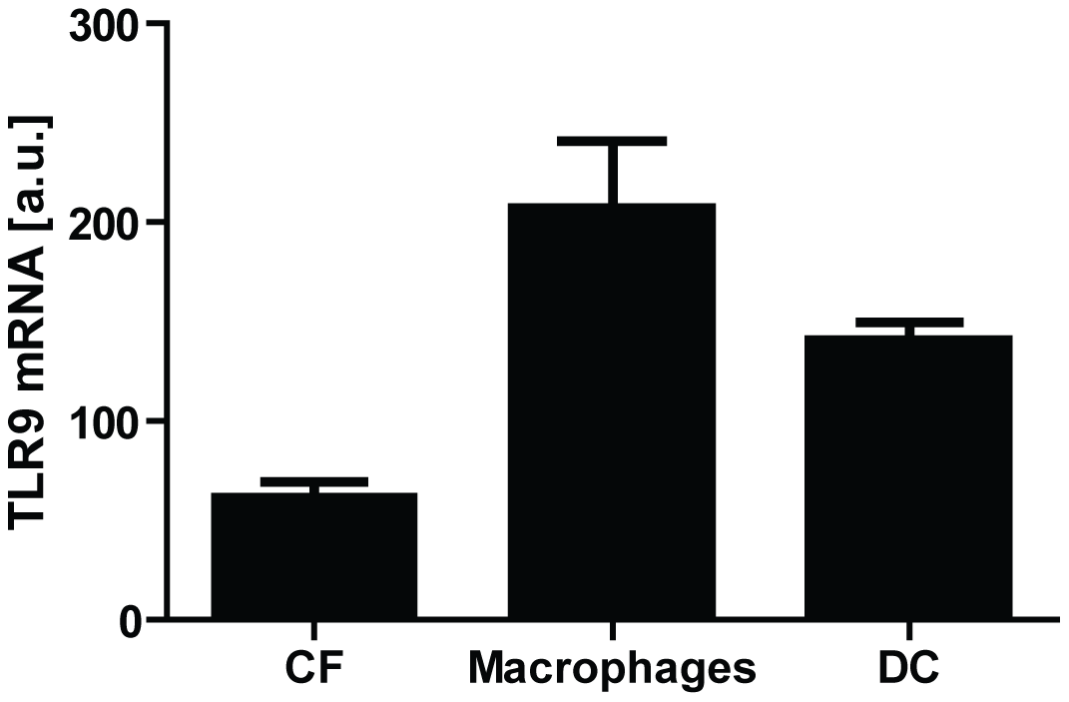
TLR9 mRNA levels in cultivated murine cardiac fibroblasts (CF; n = 3), bone marrow derived macrophages (n = 3) and dendritic cells (DC; n = 3). Data presented as mean ± SEM.

The typical responder cells in the innate immune system are monocytes/macrophages and DC. As shown in Fig. 2, only modestly higher TLR9 mRNA levels were found in macrophages and DCs, compared with that in CFs.

**Figure 3:**
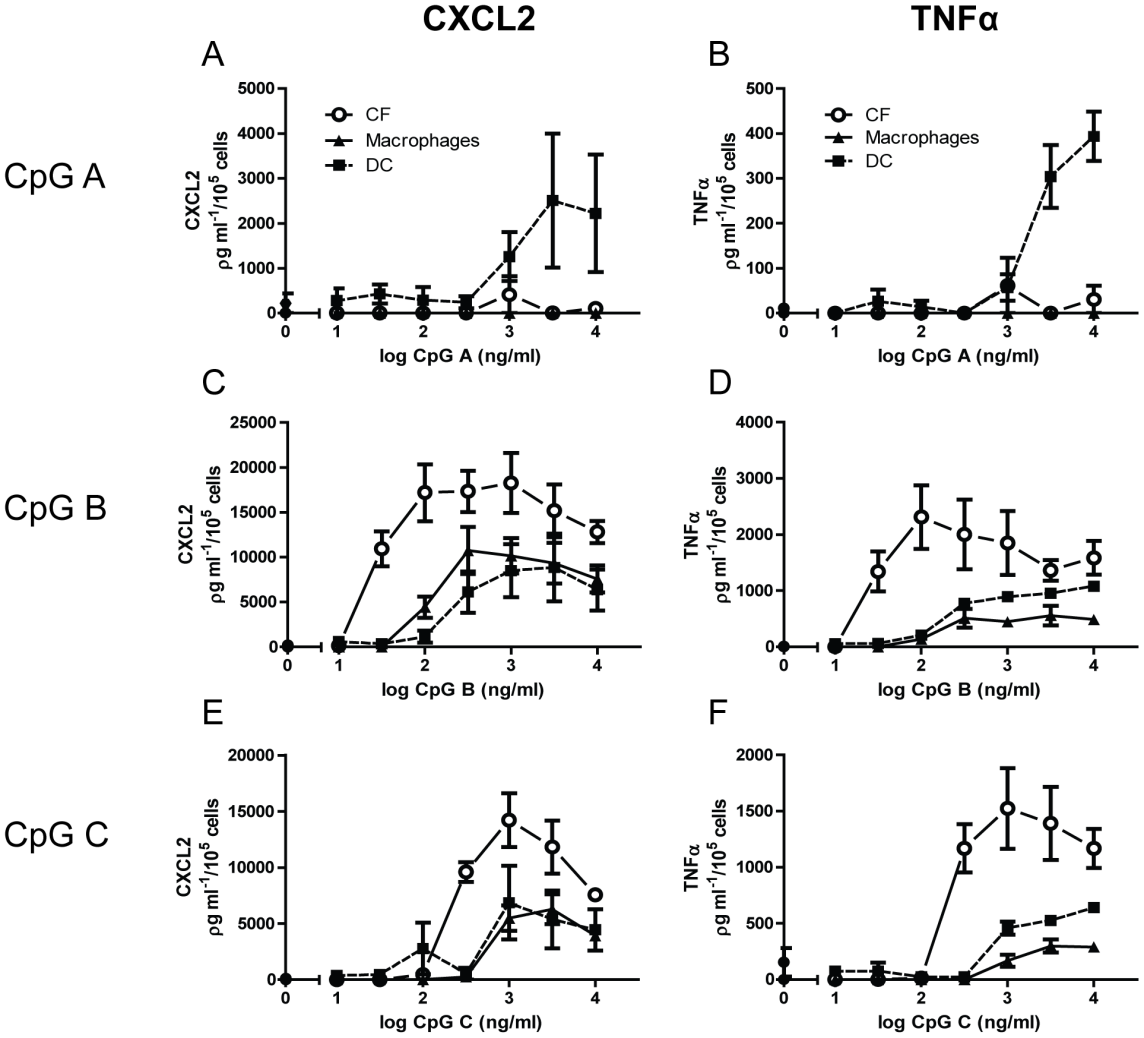
Comparative analysis of TLR9-stimulated responses between cardiac fibroblasts and bone-marrow derived -macrophages and –dendritic cells. Dose-response relationships of (100 ng/ml) CpG A (panels A–B), CpG B (panels C–D) and CpG C (panels E–F) stimulated release of CXCL2 (left panels) and TNFα (right panels) were examined in murine cardiac fibroblasts (CFs; circles) compared to bone marrow derived –macrophages (triangles) and –dendritic cells (DC; squares). Analysis was performed after 18 hours stimulation. Each data point represents the mean ± SEM of 3 experiments.

In order to evaluate the magnitude of TLR9-stimulated responses in CFs, comparing dose-response relationship experiments were undertaken in CFs and bone-marrow derived macrophages and DC of the same mice. In contrast to what seen for CFs, a clear response to CpG A could be seen in DCs (Fig. 3A–B). Surprisingly, however, a clear leftward shift of the curve for CpG B (Fig. 3C–D) and C (Fig. 3E–F), as well as higher maximal responses, could be seen in CFs as compared to DCs and macrophages, indicating higher potency and efficacy in CFs.

### Cellular functional consequences of CF TLR9 stimulation

**Figure 4:**
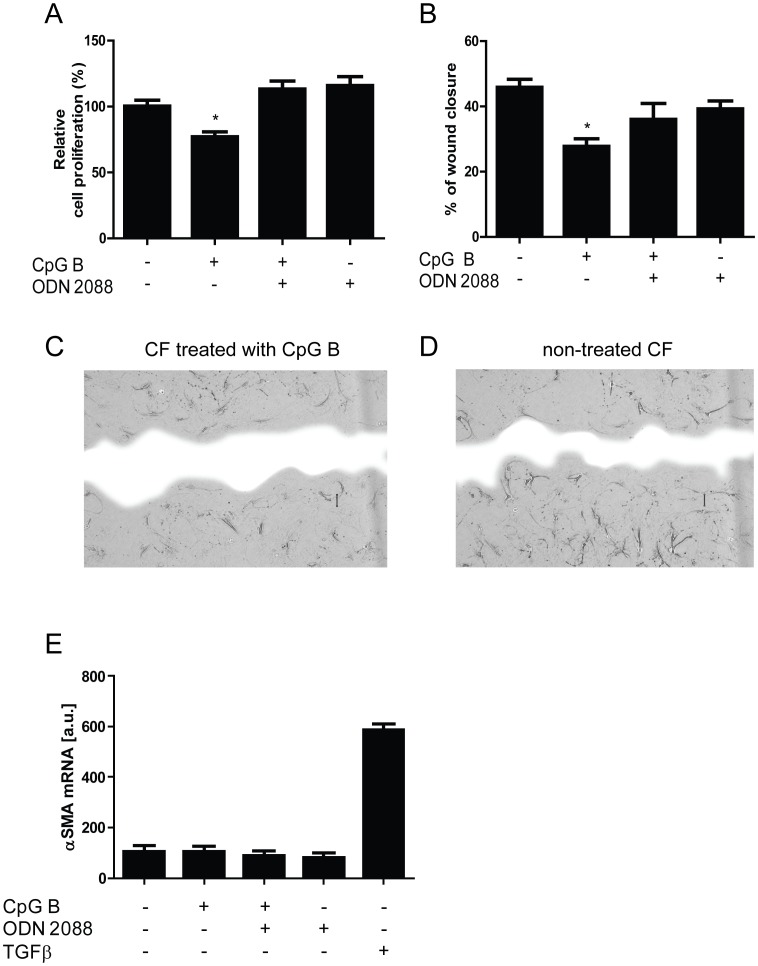
Functional analysis of TLR9-stimulated responses in cardiac fibroblasts. Proliferation by BrdU-incorporation (panel A, n = 4) and proliferation/migration by scrape wound assays (panel B n = 4) were examined in murine cardiac fibroblasts (CFs). Data are presented as mean ± SEM. *p<0.05 vs. control, **p<0.01 vs. control. Panel C and D - representative photographs of CFs proliferation/migratory responses on (100 ng/ml) CpG B stimulation (panel C) and vehicle (panel D) in scrape wound assay 18 hours after cell distortion. Blue color represents wound area. Differentiation in response to CpG B (100 ng/ml) and/or ODN 2088 (1 µg/ml) was examined in murine CFs (panel E, n = 7). TGFβ (2.5 ng/ml) serves as positive control. Data are presented as mean ± SEM.

Key features of CFs in heart diseases, including MI, are their ability to proliferate and/or migrate upon pathological stress. As depicted in Fig. 4, TLR9-stimulation by CpG B (the most potent CpG), modulates these responses as a significant attenuation of proliferation (BrdU assay, Fig. 4A) and proliferation/migration (scrape wound assay, Fig. 4B–D) was seen. Similar proliferative responses were also seen during conditions without the presence of FCS (data not shown). These responses appear to be specifically mediated through TLR9, as a significant reversal of the anti-proliferative/migratory response upon CpG B was seen with concomitant incubation with ODN 2088.

Another important feature of the CFs in MI is the ability to differentiate into a myofibroblast phenotype. This cellular event is nodal for infarct consolidation and remodeling. A key biochemical signature for this cellular transformation is the increased expression of αSMA. Twenty-four hours after subjecting CFs to serum-free DMEM, expression levels of αSMA were low. While TGFβ, a prototype-ligand promoting differentiation of CFs to myofibroblast, substantially increased αSMA-expression, similar αSMA-regulations could not be seen after stimulating TLR9 (Fig. 4E).

### Cellular origins of *in vivo* cardiac TLR9-responses

**Figure 5:**
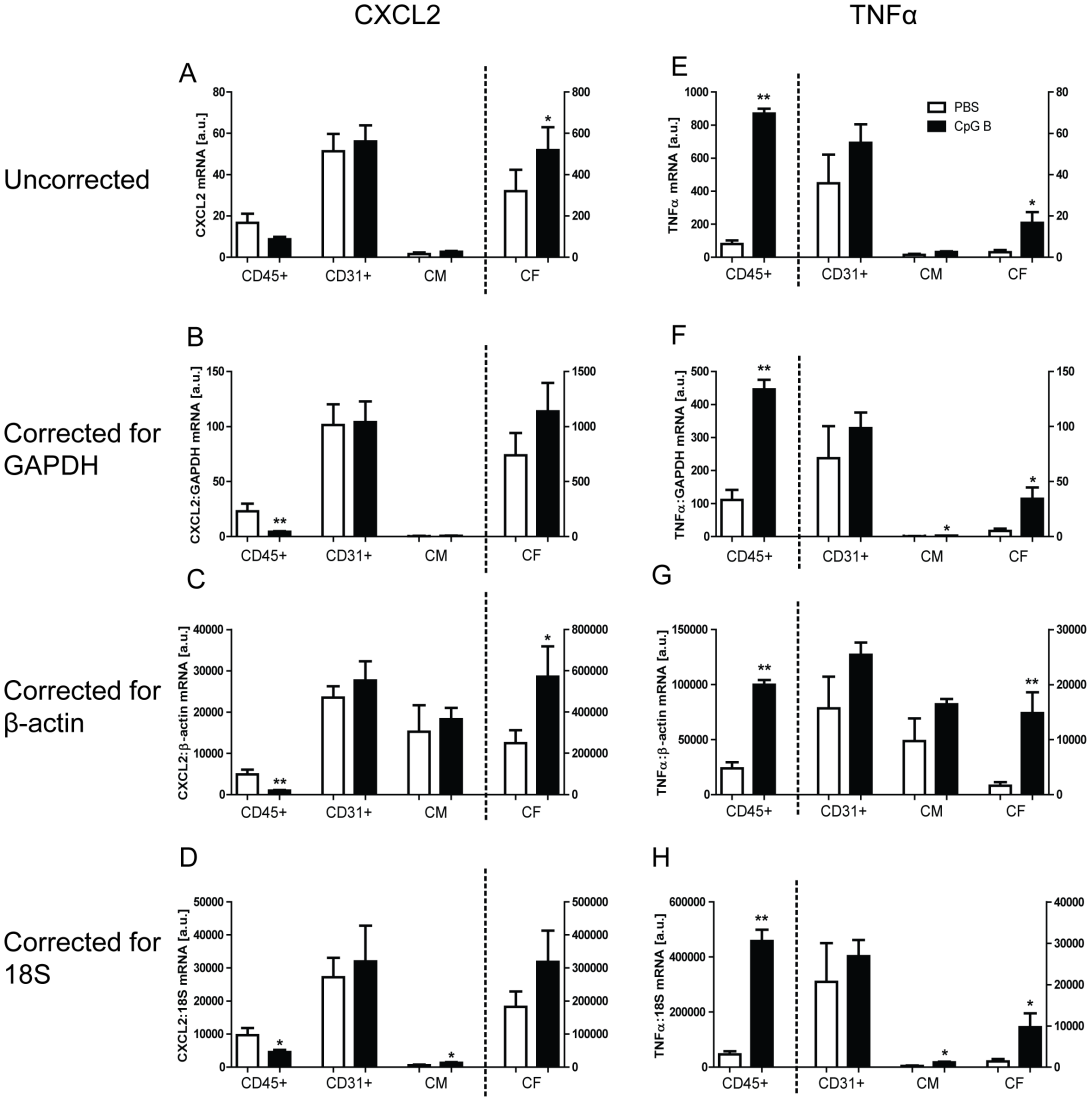
*In vivo* cardiac TLR9-stimulated cellular responses. Male C57BL/6 mice were injected i.p. with 100 µl CpG B (50 µg; n = 6, black bars) or vehicle (n = 6, white bars) and euthanized after 24 hours, with subsequent isolation of cardiac myocytes (CM), CD45^+^, CD31^+^ and non-CM/non-CD45^+^/non-CD31^+^ (denominated CF). TLR9-mediated responses in the cell-fractions were analyzed by mRNA expression levels of CXCL2 (panels A–D) and TNFα (panels E–H). Uncorrected data are shown in panels A and E. CXCL2 and TNFα are also presented as corrected for GAPDH (panels B and F), β-actin (panels C and G) and 18S (panels D and H). Data presented as mean ± SEM. *p<0.05 vs. sham, **p<0.01 vs. sham.

In order to relate our *in vitro* findings to the *in vivo* situation, mice were given i.p. injections of CpG B 24 hours prior to euthanization. Hearts were extracted, digested and subsequently specific cell-types were fractionated. Purity of the cell-fractions was found satisfactory by analyzing the expression levels of cell-specific genes; CD3γ, CD45, troponin T2, and collagen I (Fig. S1). TLR9-mediated responses were analyzed by expression levels of CXCL2 and TNFα (both known to be induced upon TLR9-stimulation). Data were analyzed uncorrected or corrected for expression levels of two housekeeping genes (GAPDH and β-actin) or 18S (Fig. 5). As for both GAPDH and β-actin, expression levels were (with the exception of the CD45^+^ fraction) not significantly different within a particular cell-fraction, but did display significantly different levels between the different cell-fractions (Fig. S2A–B). As for 18S, no significant difference could be seen between the various cell fractions, nor were there seen any significant alteration upon TLR9 stimulation (Fig. S2C). While there were no TLR9-mediated CXCL2-responses in CD31^+^ (endothelial cells) and CD45^+^ cells, and only a moderate response in CM, TLR9-activation *in vivo* appeared to induce an increase in CXCL2-expression in CFs (Fig. 5A–D). Moreover, while TLR9-activation had no effect on TNFα expression in endothelial cells and CM, it markedly increased TNFα expression in CD45^+^ cells and CFs (Fig. 5E–H). Thus, a clear induction of both CXCL2 and TNFα could be demonstrated upon TLR9–stimulation in the CF-enriched fraction, suggesting an *in vivo* relevance of our *in vitro* findings.

## Discussion

Altered TLR9-signaling has pathophysiological significance in various experimental cardiovascular disorders [4], [5], [10], [12], [13]. These studies have addressed the consequence of such receptor activity through cardiac myocytes, or the heart as a whole. However, the majority of cells in the myocardium are CFs and recent studies have attributed increasing significance of this cell-type in regards to inflammatory signaling properties [16]. In the present study we show that TLR9-activation induces potent inflammatory responses in CFs both *in vitro* and *in vivo*, and furthermore that altered CF TLR9-signaling have cellular functional consequences.

TLR9 is activated by DNA rich in unmethylated CpG-motifs, with subsequent initiation of potent inflammatory signaling [2], [4], [5], [23]. These effects can easily be mimicked by the use of different synthetic oligodeoxynucleotides [3]. Previous reports have elucidated that the subsequent choice of signaling pathway (e.g. activation of NFκB, MAPK, and/or Type I IFN) is strongly dependent on both cell-type and the nucleotide composition of the stimulating DNA [21], [22]. The three major classes of CpG ODNs we chose (class A, -B and –C), differ in respect to structure and nucleotide composition, and have previously been demonstrated to elicit different immune responses in different cell-types [21]. Whereas CpG A induce strong IFNα-response primarily in plasmacytoid DC [24] and CpG B is a strong B-cell and NFκB-activator [3], CpG C combines features of both class A and B [25].

To determine the CpG-constitution that predominates in inducing TLR9-activation in CFs, we investigated pharmacological properties, such as temporal profiles and dose-response relationships of the various CpG ODNs. Whereas we did not find any TLR9-mediated Type I IFN responses in CFs, substantial release of both CXCL2 and TNFα was found when stimulating CFs with CpG B and C, though not with CpG A. Whereas the temporal profiles of CpG B and C appeared similar, the dose-response relationships showed CpG B as the more potent of the CpG-classes tested. These findings were substantiated in similar separate experiments measuring the TLR9 stimulated transcriptional regulation of CXCL2 and TNFα (Fig. 1E–F). Furthermore, CpG B appeared to specifically activate TLR9, as both the TLR9-specfic antagonist ODN 2088 and chloroquine diphosphate completely blocked responses. Thus, our data demonstrate CFs to express functional TLR9. Our findings of CpG class B being the more potent on CF-mediated TLR9-responses, differs from previously published data on CM. Mathur *et al* demonstrated that CpG class C led to increased NFκB-activity in CM, whereas CpG A and B had minimal biological activity [12]. Of note, unpublished data from our group recapitulated these results. The observed differences between CpG ODN stimulation of CFs and CM suggest that TLR9-responses in the myocardium are cell-specific.

In order to evaluate the magnitude of the TLR9-stimulated responses in CFs, we compared the secretory responses following CpG ODN-stimulation with that of classical innate immune cells, such as macrophages and DC. Prior to executing these experiments, we analyzed mRNA expression levels of TLR9 in these cultivated cells. As shown in Fig. 3, TLR9 mRNA was only modestly higher in cultivated immune cells compared with CFs. As expected, and in accordance to previous publications [24], TLR9-activation by CpG A was seen in DC, but not in macrophages or CFs. However, both CpG B and C induced a more potent TLR9-response in CFs compared to either of the innate immune cells. Even though these results appeared consistent throughout three separate experiments (n = 3), our low sample size is a limitation as to the conclusions. *In vitro* assessment of receptor-stimulated responses should be interpreted with caution as to *in vivo* extrapolation. However, the robust responses seen upon TLR9-stimulation in CFs suggest that CFs may contribute to TLR9-mediated responses in the myocardium.

While TLR9 promote inflammatory responses in CFs, the consequence of TLR9 stimulation as to classical CF-properties remains unknown and unaddressed. TLR9-activation has been shown to stimulate invasion in glioblastoma, astrocytoma and breast cancer epithelial cells [26], and also cause differentiation of pulmonary fibroblasts into a myofibroblast phenotype [27]. Thus, according to the above-mentioned studies, TLR9-stimulation appears to activate migration/proliferation and induce differentiation. In contrast, our *in vitro* data demonstrate that stimulation of TLR9 attenuates proliferation and migration in CFs. These functional responses were proven TLR9-specific as concomitant treatment with the TLR9-antagonist (ODN 2088) reversed the inhibiting effect. Moreover, TLR9-stimulation did not influence differentiation of CFs into myofibroblasts, as the expression of αSMA was unchanged after 24-hour TLR9 stimulation. This observation is in accordance with previously published findings, in which CpG ODN priming of TLR9 attenuated collagen deposition following transverse aortic constriction, by inhibiting the transdifferentiation of CFs to myofibroblasts [13].

In order to substantiate our *in vitro* conclusions of CFs being a robust TLR9-responsive cell-type, we conducted *in vivo* experiments where specific cardiac cells were isolated 24-hours after initial TLR9-stimulation. To our knowledge, this is the first study to explore *in vivo* cardiac cell-specific TLR9-responses. A prerequisite for valid conclusions in this experiment is a high degree of cell-specific fractionation. As demonstrated in the Fig. S1, analysis of mRNA expression levels of genes reported to be highly cell-specific allowed us to conclude that our method was satisfactory. As a method for studying cell-specific *in vivo* activation of TLR9-signaling pathways, we analyzed mRNA expression levels of CXCL2 and TNFα. As expected, the basal levels of these cytokines differed substantially between the different cell-fractions, this most likely being the product of numerous inflammatory signaling pathways leading to NFκB activation. However, in agreement with our *in vitro* data we observed a significant induction above basal levels of both CXCL2 and TNFα upon TLR9-stimulation, in the CF-enriched cell-fraction. A limitation to the latter conclusion is that the significant induction of CXCL2 seen upon analysis of uncorrected data, as well as corrected for β-actin, were not found when correcting for GAPDH or 18S. For the TLR9-stimulated induction of TNFα, this was found significant regardless of whether uncorrected or corrected. Being the more numerous of the cardiac cell-types, such induction suggests that CFs are capable (given the presence of a significant endogenous TLR9 ligand) of contributing a substantial portion of the cardiac TLR9-stimulated inflammatory responses. In addition, and also in agreement with our *in vitro* data, the observed differentiated responses to TLR9-stimulation in the CM fraction, as well as the differences in TLR9-responses in the CD45^+^ and CD31^+^ cell-fractions, supports the notion of differentiated, cell-type specific TLR9-stimulated responses within the myocardium.

We did not investigate the functional and pathophysiological significance of our findings, nor did we address the significance of putative cardiac endogenous cardiac TLR9-ligands. However, evidence from other groups, as well as ours, has suggested released mitochondrial DNA as a TLR9-ligand [4], [5], [28]. There are contrasting results regarding the consequence of TLR9-stimulation in heart failure. Whereas Oka *et al* demonstrated that increased TLR9 signaling restricted to the CM is detrimental in murine pressure-overload cardiomyopathy [5], Velten *et al* demonstrated the opposite in the same experimental model upon systemic administration of a TLR9-agonist [13]. Although there may be many differences between these two approaches as to explain the striking discrepancy in outcome, one difference is TLR9-signaling restricted to the CM versus TLR9-stimulation of all cardiac TLR9 responder cells. Indeed, our results in the present study suggest that future studies have to consider TLR9-signaling through cardiac cells other than the CM.

CFs constitute nodal cells within the myocardium and are important in cardiac remodeling upon heart disease [15]. This study is predominantly an *in vitro* conducted study, and thus conclusions regarding *in vivo* consequences are speculative. However, our findings may suggest a sentinel role for CFs in promoting cardiac TLR9-responses. This should be considered in future studies on the pathophysiological significance of TLR9 in cardiovascular diseases.

## Supporting Information

Figure S1 **Purity of cell fractions.** Male C57BL/6 mice were injected i.p. with 100 µl CpG B (50 µg; n = 6) or vehicle (n = 6) and euthanized after 24 hours, with subsequent isolation of cardiac myocytes (CM), CD45^+^, CD31^+^ and non-CM/non-CD45^+^/non-CD31^+^ (denominated CF). Purity of the isolated cell fractions were determined by analyzing the expression levels of CD3γ, CD45, tronponin T2 and collagen I by real time-PCR. Data presented as mean ± SEM.
(TIF)

Figure S2 **Expression levels of housekeeping genes and 18S in separated cardiac cell fractions.** The separated murine cardiac cell fractions: CD45^+^ cells, CD31^+^ cells, cardiac myocytes (CM) and cardiac fibroblasts (CF) were analyzed for the housekeeping genes GAPDH (panel A) and β-actin (panel B), as well as 18S (panel C) by real-time PCR. Open circles: CpG B injected (n = 6), black squares (n = 6): vehicle injected. Data presented as mean ± SEM.
(TIF)
