# Supplementary figures and images for: Toll-Like Receptor 9 Mediated Responses in Cardiac Fibroblasts
Source: PLoS One. 2014 Aug 15;9(8):e104398. doi: 10.1371/journal.pone.0104398 (PMC4134207; doi:10.1371/journal.pone.0104398)

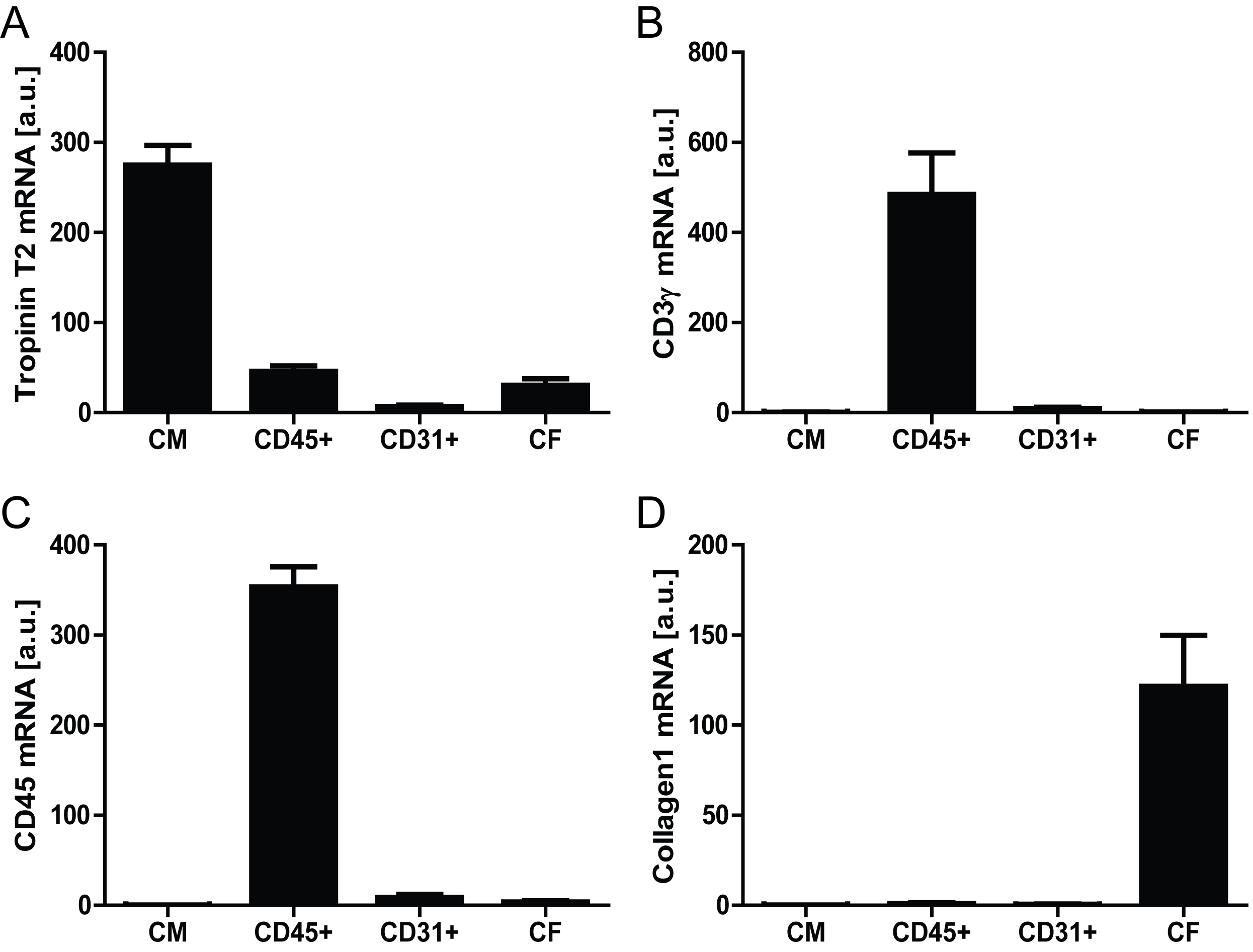

Supplement: Figure S1 — Purity of cell fractions. Male C57BL/6 mice were injected i.p. with 100 µl CpG B (50 µg; n = 6) or vehicle (n = 6) and euthanized after 24 hours, with subsequent isolation of cardiac myocytes (CM), CD45+, CD31+ and non-CM/non-CD45+/non-CD31+ (denominated CF). Purity of the isolated cell fractions were determined by analyzing the expression levels of CD3γ, CD45, tronponin T2 and collagen I by real time-PCR. Data presented as mean ± SEM. (TIF) [file pone.0104398.s001.tif]

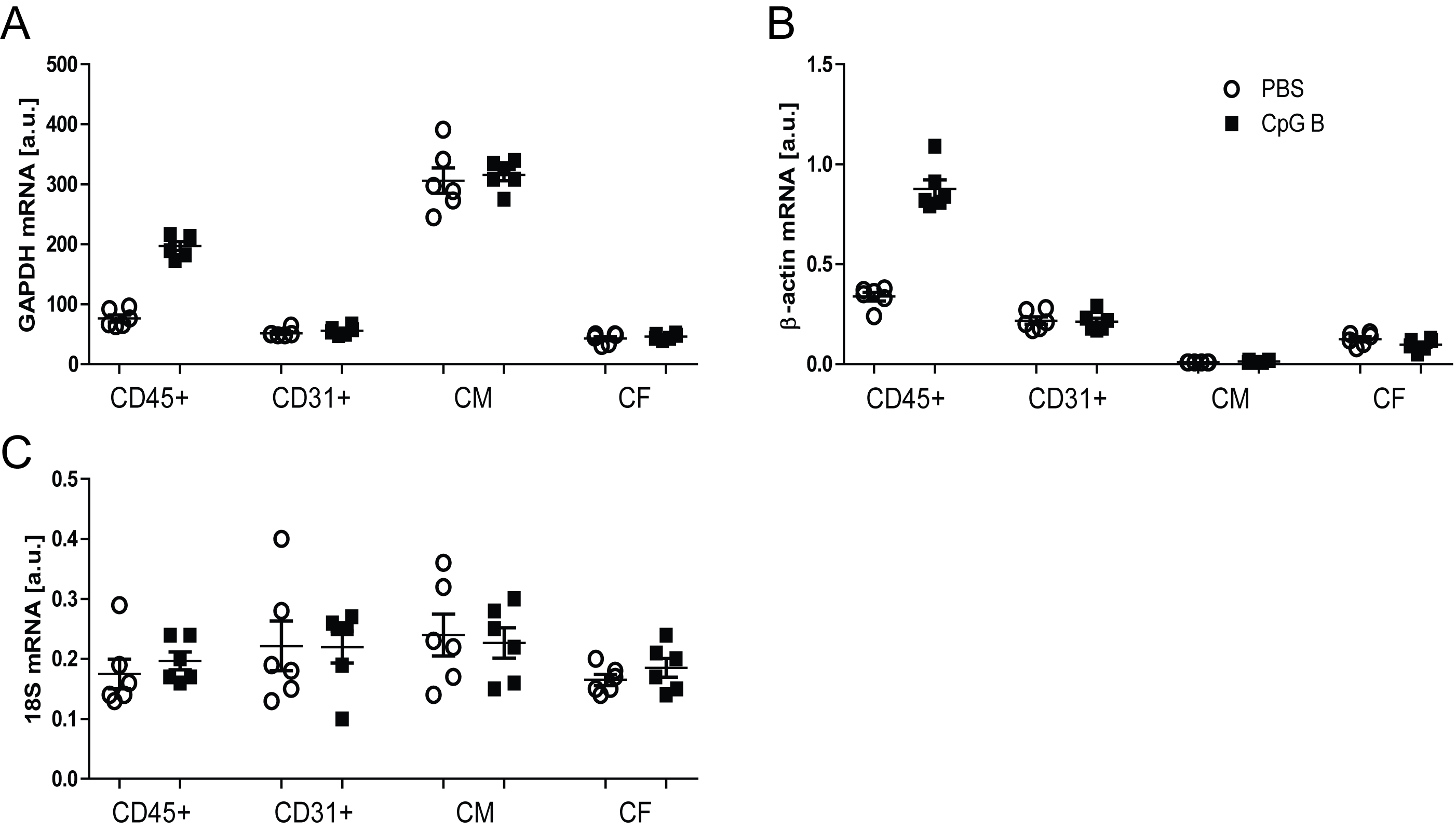

Supplement: Figure S2 — Expression levels of housekeeping genes and 18S in separated cardiac cell fractions. The separated murine cardiac cell fractions: CD45+ cells, CD31+ cells, cardiac myocytes (CM) and cardiac fibroblasts (CF) were analyzed for the housekeeping genes GAPDH (panel A) and β-actin (panel B), as well as 18S (panel C) by real-time PCR. Open circles: CpG B injected (n = 6), black squares (n = 6): vehicle injected. Data presented as mean ± SEM. (TIF) [file pone.0104398.s002.tif]
